# Synergistic Targeting of DNA-PK and KIT Signaling Pathways in KIT Mutant Acute Myeloid Leukemia

**DOI:** 10.1016/j.mcpro.2023.100503

**Published:** 2023-01-20

**Authors:** Heather C. Murray, Kasey Miller, Joshua S. Brzozowski, Richard G.S. Kahl, Nathan D. Smith, Sean J. Humphrey, Matthew D. Dun, Nicole M. Verrills

**Affiliations:** 1 School of Biomedical Sciences and Pharmacy, College of Health, Medicine and Wellbeing, University of Newcastle, and Hunter Cancer Research Alliance and Precision Medicine Program, Hunter Medical Research Institute, Callaghan, New South Wales, Australia; 2 Analytical and Biomolecular Research Facility, Advanced Mass Spectrometry Unit, University of Newcastle, Callaghan, New South Wales, Australia; 3 School of Life and Environmental Sciences, and The Charles Perkins Centre, The University of Sydney, Sydney, New South Wales, Australia

**Keywords:** AML, c-KIT, DNA-PK, phosphoproteomics, synergistic targeted therapies, leukaemia, AKT, RAC-alpha serine/threonine-protein kinase, AML, acute myeloid leukemia, BTK, Bruton’s tyrosine kinase, DDA, data-dependent acquisition, DNA-PK, DNA-dependent protein kinase, ERK, extracellular signal–regulated kinase, EV, empty vector, FLT3, receptor-type tyrosine-protein kinase FLT3, FYN, tyrosine-protein kinase Fyn, GRB2, growth factor receptor–bound protein 2, JAK, tyrosine-protein kinase JAK, KIT, mast/stem cell growth factor receptor kit, LYN, tyrosine-protein kinase Lyn, MAPK, mitogen-activated protein kinase, MTOR, serine/threonine-protein kinase mTOR, PRM, parallel reaction monitoring, ROS, reactive oxygen species, RTK, receptor tyrosine kinase, STAT, signal transducer and activator of transcription, TKI, tyrosine kinase inhibitor

## Abstract

Acute myeloid leukemia (AML) is the most common and aggressive form of acute leukemia, with a 5-year survival rate of just 24%. Over a third of all AML patients harbor activating mutations in kinases, such as the receptor tyrosine kinases *FLT3* (receptor-type tyrosine-protein kinase FLT3) and *KIT* (mast/stem cell growth factor receptor kit). *FLT3* and *KIT* mutations are associated with poor clinical outcomes and lower remission rates in response to standard-of-care chemotherapy. We have recently identified that the core kinase of the non-homologous end joining DNA repair pathway, DNA-PK (DNA-dependent protein kinase), is activated downstream of FLT3; and targeting DNA-PK sensitized *FLT3*-mutant AML cells to standard-of-care therapies. Herein, we investigated DNA-PK as a possible therapeutic vulnerability in *KIT* mutant AML, using isogenic FDC-P1 mouse myeloid progenitor cell lines transduced with oncogenic mutant *KIT* (V560G and D816V) or vector control. Targeted quantitative phosphoproteomic profiling identified phosphorylation of DNA-PK in the T2599/T2605/S2608/S2610 cluster in *KIT* mutant cells, indicative of DNA-PK activation. Accordingly, proliferation assays revealed that *KIT* mutant FDC-P1 cells were more sensitive to the DNA-PK inhibitors M3814 or NU7441, compared with empty vector controls. DNA-PK inhibition combined with inhibition of KIT signaling using the kinase inhibitors dasatinib or ibrutinib, or the protein phosphatase 2A activators FTY720 or AAL(S), led to synergistic cell death. Global phosphoproteomic analysis of KIT-D816V cells revealed that dasatinib and M3814 single-agent treatments inhibited extracellular signal–regulated kinase and AKT (RAC-alpha serine/threonine-protein kinase)/MTOR (serine/threonine-protein kinase mTOR) activity, with greater inhibition of both pathways when used in combination. Combined dasatinib and M3814 treatment also synergistically inhibited phosphorylation of the transcriptional regulators MYC and MYB. This study provides insight into the oncogenic pathways regulated by DNA-PK beyond its canonical role in DNA repair and demonstrates that DNA-PK is a promising therapeutic target for *KIT* mutant cancers.

## Highlights

• DNA-PK is phosphorylated (activated) in mutant-KIT cells.
• Phosphoproteomic characterization of oncogenic mutant D816V-KIT cells.
• Mutant-KIT cells are sensitive to DNA-PK inhibitors.
• DNA-PK inhibitors synergize with KIT signaling inhibitors in mutant-KIT cells.
• Combined DNA-PK and KIT inhibition reduced ERK, MTOR, MYC, and MYB phosphorylation.

## Graphical Abstract

**Figure:**
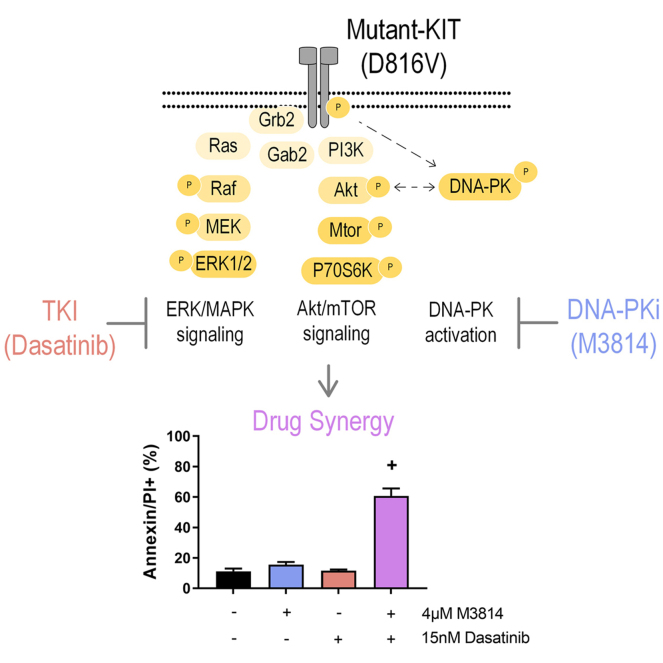

## In Brief

This study provides insight into the oncogenic pathways regulated by KIT, revealing an important role for DNA-PK activation, beyond its canonical role in DNA repair. DNA-PK activation may be a common event in receptor tyrosine kinase mutant AML and is a promising novel therapeutic target for this poor-prognosis AML subtype.

Acute myeloid leukemia (AML) arises from deregulated proliferation of myeloid progenitor cells, culminating in an overpopulation of leukemic “blast” cells in the bone marrow and other tissues. AML is the most lethal form of leukemia, accounting for less than one-third of all diagnoses but nearly two-thirds of leukemia deaths (1). Since 1973, the mainstay of therapy for AML has been the cytotoxic chemotherapy “7 + 3” regimen; 7 days infusion of the antimetabolite cytarabine, combined with an intravenous anthracycline for the first 3 days (2). Approximately 60% of patients achieve primary remission with this regimen (3, 4); however, the majority relapse. Recent advances have seen the introduction of targeted therapies; however, the frequent and rapid development of resistance has limited their therapeutic benefit in AML (5, 6). Five-year survival for AML is just 24% (1), highlighting the need for less toxic and more effective treatments.

Approximately 50% of AML patients harbor mutations in genes encoding kinases or phosphatases (7). Commonly mutated kinases include receptor-type tyrosine-protein kinase FLT3 (*FLT3*) (22–30% of patients) and mast/stem cell growth factor receptor kit (*KIT*) (4–14% of patients) (7, 8, 9). These mutations lead to constitutively active kinase signaling and rarely co-occur (7, 9). KIT (CD117) is a class III receptor tyrosine kinase (RTK), sharing high sequence similarity with the other class III RTK family members, FLT3 and platelet-derived growth factor receptor (10, 11). KIT contains five immunoglobulin-like extracellular domains, a transmembrane domain, and intracellular juxtamembranous and split kinase domains. Wildtype KIT receptor activity is regulated by its endogenous ligand, stem cell factor. Stem cell factor binding leads to KIT receptor dimerization (12), stimulating transautophosphorylation (13). Docking and subsequent phosphorylation of signaling mediators such as FYN (tyrosine-protein kinase Fyn) (14), LYN (tyrosine-protein kinase Lyn) (15), signal transducer and activator of transcription 1 (STAT1) (16), and growth factor receptor–bound protein 2 (GRB2) (17) activates downstream networks including PI3K/AKT (RAC-alpha serine/threonine-protein kinase) (14, 18), mitogen-activated protein kinase (MAPK) (18), and JAK (tyrosine-protein kinase JAK)/STAT (19).

Approximately 80% of AMLs express KIT (20), and high expression is associated with poor prognosis (21, 22). KIT-activating mutations occur in a range of neoplasms, including AML (23), systemic mastocytosis (24), melanoma (25), and gastrointestinal stromal tumors (26). *KIT* mutations in AML are most common in the core-binding factor (CBF) subtype, occurring in 25 to 40% of CBF-AML patients (27, 28). The most frequent *KIT* mutations are point mutations in codon 816 within the second tyrosine kinase domain (29), most often leading to a valine substitution for aspartic acid (D816V) (30, 31). Although CBF-AML has a relatively favorable prognosis (32), the presence of mutant *KIT* is associated with a lower complete remission rate (33) and reduced survival (34, 35).

Similarly to activating mutations of FLT3 (36), KIT-activating mutations in the tyrosine kinase domain (D816V) or juxtamembrane domain (V560G) lead to elevated intracellular reactive oxygen species (ROS) (37). At high levels, intracellular ROS induces DNA damage, including oxidized bases and DNA double-strand breaks (36, 38). Elevated DNA double-strand break repair activity could enable cell survival in the presence of increased ROS, presenting a potential therapeutic vulnerability. Indeed, oncogenic *FLT3* mutations induce activation of the non-homologous end joining DNA repair pathway (39, 40). We have recently shown that this is associated with increased phosphorylation of the nonhomologous end joining core kinase, DNA-PK; and importantly, that *FLT3*-mutant cells are sensitive to pharmacological DNA-PK inhibition (41).

Herein, we show that expression of D816V- and V560G-KIT mutations is associated with increased DNA-PK phosphorylation, consistent with DNA-PK activation. Accordingly, mutant D816V and V560G-KIT cell lines are sensitive to DNA-PK inhibitors, and DNA-PK inhibition combined with KIT signaling inhibition induces synergistic cell death. Mechanistically, discovery phosphoproteomic analysis of combined DNA-PK and tyrosine kinase inhibitor (TKI) treatment in D816V-KIT cells revealed co-operative inhibition of AKT/mTOR, P70S6K, and extracellular signal–regulated kinase (ERK)/MAPK signaling, and synergistic inhibition of phosphorylation of the transcription factors MYC and MYB.

## Experimental Procedures

### Cell Lines

Mouse myeloid progenitor FDC-P1 cells transduced with mutant *KIT* (D816V or V560G) or an empty vector (EV) were generated previously (42). All FDC-P1 cell lines were maintained in standard culture conditions (37 °C, 5% CO_2_), in Dulbecco's modified Eagle's medium containing 10% fetal calf serum, 20 mM Hepes, and 2 mM l-glutamine. EV cell lines were expanded with 0.5 ng/ml mouse granulocyte macrophage-colony stimulating factor (BioLegend). All lines were routinely confirmed to be free of mycoplasma contamination using the MycoAlert mycoplasma detection kit (Lonza), as per the manufacturer’s instructions. KIT expression was monitored using flow cytometry (Supplementary Methods).

### Drugs

Cell lines were treated with the following agents, either alone or in combination as indicated in text. DNA-PK inhibitors: NU7441 (Selleckchem), M3814 (Merck); KIT signaling inhibitors: dasatinib (Cayman Chemical), ibrutinib (Selleckchem), FTY720 (Cayman Chemical), and AAL(S) (synthesized by A/Prof Jonathan Morris, School of Chemistry, UNSW as described (43)). Dimethyl sulfoxide was used as the solvent for all compounds. Final vehicle concentration was below 0.1% for all experiments.

### Cell Viability and Apoptosis Analysis

Cell viability in response to 72 h drug treatments was assessed using the metabolic resazurin assay, as previously described (41). Apoptotic cells were measured using annexin V-FITC or annexin V-APC flow cytometry assays (BD Biosciences), as per the manufacturer’s instructions. For combination drug treatments, synergy was assessed using the fractional product method of Webb (44). Graphs were produced using GraphPad Prism 9 software (GraphPad Software, Inc).

### Synergy Analysis

For combination drug treatments, synergy was evaluated using the method of Webb (also termed the “fractional product method”) (44). The method of Webb estimates the expected additive effect of two drugs using the fractional product of the effect of each drug alone, that is:

1−Fa(Drug1+Drug2)=(1−FaDrug1)∗(1−FaDrug2)

Where Fa = the fraction of cells affected, expressed as a decimal.

An observed Fa(Drug1 + Drug2) value greater than the expected Fa(Drug1 + Drug2) value indicates synergy, whereas an observed Fa(Drug1 + Drug2) lower than the expected value indicates antagonism. The Webb result was calculated by subtracting the observed Fa(Drug1 + Drug2) from the expected Fa(Drug1 + Drug2). A result less than −0.1 was defined as synergistic.

Where dose–response curves were obtained, synergy was also assessed using the model of Bliss (45), evaluated using SynergyFinder (46). A Bliss result greater than 10 was defined as synergistic.

### Phosphoproteomic Profiling

Label-free global phosphoproteomic profiling was performed using the EasyPhos method (47). In brief, 200 μg of lysate was reduced and alkylated using Tris(2-carboxyethyl)phosphine hydrochloride and 2-chloroacetamide. Lysates were digested using trypsin-LysC (V5072; Promega), overnight at 37 °C with shaking (1500 rpm). Phosphopeptides were subsequently enriched using TiO_2_ beads and desalted using SDB-RPS StageTips. The nonmodified proteome flow through was desalted using ethylacetate phase separation and desalted using SDB-RPS StageTips using a custom 3D-printed adapter (48).

Peptides were further purified with an Acclaim PepMap 100 C18 75 μM ✕ 20 mm trap column (Thermo Fisher) prior to separation on a 75 μM ✕ 25 cm EASY-Spray PepMap C18 column (Thermo Fisher) using a 5–35% acetonitrile gradient on an Exploris 480 mass spectrometer (Thermo Fisher). A FAIMSpro compensation voltage of −60 was used. For data-dependent acquisition (DDA) analysis, peptides were separated using a 90 min gradient. Full MS scans of 350 to 1200 *m/z* were acquired at a resolution of 120,000, with an automatic gain control of 3e6 and maximum injection time of 50 ms. MS/MS scans were acquired using a resolution of 45,000, automatic gain control of 2.5e6, a normalized collision energy of 36, and maximum injection time of 100 ms. For targeted analyses using parallel reaction monitoring (PRM) (49), an Exploris 480 or Oribtrap Eclipse mass spectrometer was used, with a FAIMS compensation voltage of −60. For the Exploris 480, peptides were separated using a 60 min gradient. Full MS scans of 370 to 1500 *m/z* were acquired with resolution 60,000, an automatic gain control of 1e6, and maximum injection time of 50 ms. MS/MS scans were acquired using resolution 15,000, an automatic gain control of 1e6, and maximum injection time of 120 ms. For the Orbitrap Eclipse, peptides were purified with a PepMap Neo trap column (Thermo Fisher) prior to separation on a 75 μM ✕ 15 cm EASY-Spray PepMap Neo column (Thermo Fisher) using a 70 min gradient. Full MS scans of 370 to 1500 *m/z* were acquired with resolution 60,000, an automatic gain control of 100%, and maximum injection time 50 ms. MS/MS scans were acquired using resolution 15,000, an automatic gain control of 200%, and maximum injection time of 120 ms.

DDA data were analyzed using Proteome Discoverer 2.5 (Thermo Fisher) as described (50). Spectrum files were recalibrated using the PD node “Spectrum files RC.” SEQUEST HT was used to search against the UniProt Mus Musculus proteome (25,342 sequences, downloaded September 27, 2021). Search parameters included a precursor mass tolerance of 10 ppm, a fragment mass tolerance of 0.02 Da, and trypsin digestion with up to two missed cleavages allowed. Cysteine carbamidomethylation was set as a fixed modification, whereas dynamic modifications were phosphorylation (S/T/Y), acetylation (N terminus, K), oxidation (M), deamidation (N/Q), and N-terminal methionine loss. A site probability threshold of 75/100 was used. Percolator (51) was used to filter the results to a 1% false discovery rate at the peptide level, using the target-decoy strategy. Label-free quantification was performed using “Minora Feature Detector,” “Feature Mapper,” and “Precursor Ions Quantifier” nodes as described (52). Minora Feature Detector detects chromatographic peaks and maps them to peptide spectral matches. Feature Mapper performs retention time alignment and links features across all raw files. The Precursor Ions Quantifier performs label-free precursor ion quantification. *t* Tests were performed using the background-based method in Proteome Discoverer 2.5 (50). The peptide list was refined to include only entries with quantitative values in all replicates of at least one treatment group. Where duplicate peptides with the same phosphosite(s) were identified, the peptide with the highest fold change was used.

Significant (*p* < 0.05) phosphosite changes with a fold change greater than ±1.5 were subject to pathway enrichment analysis using ingenuity pathway analysis (QIAGEN). Enriched kinases were identified by mapping the upstream kinase(s) for each identified phosphosite (supplemental Table S2) using both mouse and human annotated kinase–substrate relationships in the Phosphosite Plus database (53). Kinases with more than five substrates with significantly increased phosphorylation, or more than five substrates with significantly decreased phosphorylation, were classified as enriched. This analysis was complemented with kinase substrate enrichment analysis using RoKAI (54), wherein data were searched against the PhosphositePlus (53) and DEPOD databases (55). Results were filtered for significantly enriched kinases and phosphatases with five or more mapped substrates.

### Development and Analytical Validation Targeted MS Assays/Measurements

We performed tier 3 PRM analysis. To identify the optimal precursor *m/z*, the targets for PRM analysis were selected from public and in-house DDA data. PRM data files were analyzed using Skyline 20.2 (MacCoss Lab). The digestion enzyme was set to trypsin, precursor charges 2 to 4 were allowed, and ion charges 1 to 3 were allowed. Transitions were selected based on coelution and precursor and fragment ion mass error. Peptide abundance was assessed by summing the peak area of the four to six highest intensity fragment ions. Results were normalized to the total ion current total area. For DNA-PKC total protein measurements, the quantification values of the three peptides (supplemental Table S1) were averaged. Statistical analysis was performed using GraphPad Prism, version 9 for Windows, using one-way ANOVA with Tukey’s or Dunnett’s adjustment for multiple comparisons. An adjusted *p* value <0.05 was considered significant.

### Western Blot

Whole-cell lysates were sonicated in ice-cold radioimmunoprecipitation assay buffer freshly supplemented with protease inhibitors (0.05 M Hepes [pH 7.4], 1% Triton X-100, 0.1% SDS, 50 mM sodium fluoride, 0.05 M EDTA, 5% sodium deoxycholate, 1 mM sodium orthovanadate, and Protease Inhibitor Cocktail [Sigma]). Total protein was quantified by bicinchoninic acid assay, as per the manufacturer’s instructions (Thermo Scientific).

Lysates were resolved in reducing conditions on 4 to 12% gradient Bis–Tris NuPAGE precast gels (Thermo Scientific) before wet transfer to nitrocellulose. Blots were probed with primary antibodies MTOR (4517S), pMTOR S2448 (5536S), Bruton’s tyrosine kinase (BTK) (8547S), pBTK Y223 (5082S), ERK1/2 (9107S), or pERK1/2 T202/Y204 (mouse T203/Y205, 9101S) (Cell Signaling Technology) followed by horseradish peroxidase–conjugated mouse or rabbit secondary antibody. Blots were developed using Immobilon Classico ECL substrate (Millipore) before visualization on a Chemidoc imager (Bio-Rad). Densitometry was performed using Image Lab (Bio-Rad).

### Experimental Design and Statistical Rationale

Proteomic analysis was performed with n = 3 biological replicates per group. For DDA data, the peptide list was refined to include only entries with quantitative values in all replicates of at least one treatment group. Differentially expressed proteins were defined by a fold change ≥1.5 or ≤−1.5 and *p* value ≤0.05. Statistical analysis for DDA data was performed using Proteome Discoverer, version 2.5. For all other experiments, the number of biological replicates used in each experiment are presented in the figure legends. Graphs were prepared using GraphPad Prism, version 9 for Windows and are presented as mean values ± SEM.

Figures were produced using Microsoft Excel and GraphPad Prism 9.

## Results

### Mutational Activation of KIT Increases DNA-PK Phosphorylation

**Fig. 1:**
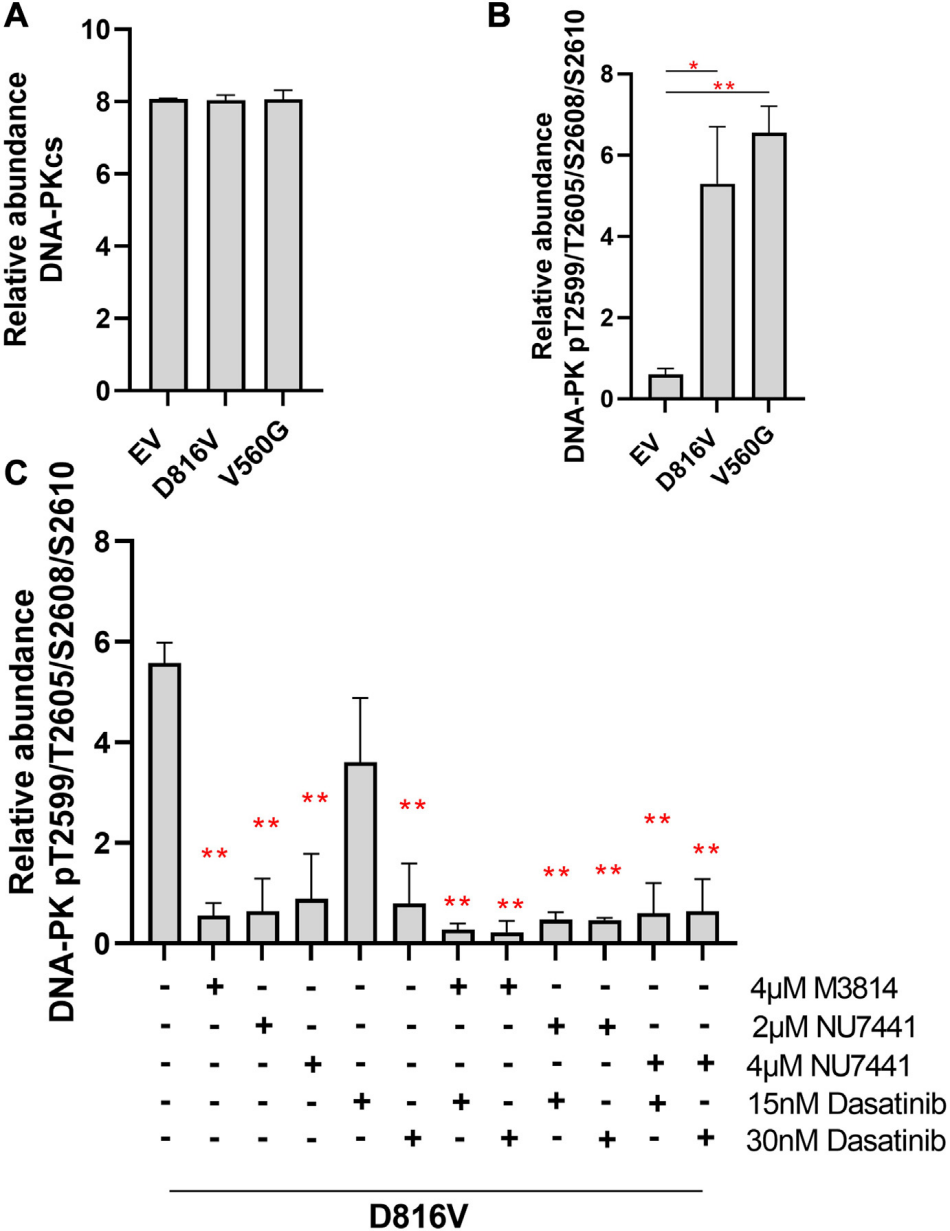
**Activated KIT signaling increases DNA-PKphosphorylation.***A*, protein expression. *B*, phosphorylation of DNA-PKcs, assessed by parallel reaction monitoring (PRM) mass spectrometry, in FDC-P1 cells transduced with an empty vector (EV) or factor-independent mutant forms of KIT (V560G and D816V). N = 3 per experiment. ∗*p* < 0.05, ∗∗*p* < 0.01 (ANOVA + Tukey’s test). *C*, phosphorylation of DNA-PK, assessed by PRM mass spectrometry, in FDC-P1/D816V-KIT cells untreated or treated for 1 h with 4 μM M3814, 2 μM NU7441, 4 μM NU7441, 15 nM dasatinib, 30 nM dasatinib, or their combinations. N ≥ 2. ∗*p* < 0.05, ∗∗*p* < 0.01 (ANOVA + Dunnett’s test). A singly phosphorylated DNA-PK peptide from the T2599/T2605/S2608/S2610 cluster was identified; however, the phosphorylation could not be localized to a specific site. DNA-PK, DNA-dependent protein kinase; cs, catalytic subunit; KIT, mast/stem cell growth factor receptor kit.

To investigate if expression or phosphorylation of DNA-PK is associated with KIT activity, we used an established FDC-P1 isogenic myeloid cell line model of oncogenic mutant constitutively active KIT signaling (56). PRM revealed that FDC-P1 cells transduced with V560G-*KIT*, D816V-*KIT*, and EV controls expressed an equal level of the catalytic subunit of DNA-PK, DNA-PKcs (Fig. 1*A*). In contrast, the phosphorylation of DNA-PKcs in the T2599/T2605/S2608/S2610 cluster was significantly higher in cell lines expressing mutant KIT (V560G and D816V) compared with the EV control (Fig. 1*B*), suggesting DNA-PK activity is elevated in cells with constitutively active mutant KIT. As expected, acute (1 h) treatment of D816V-KIT cells with DNA-PK inhibitors M3814 or NU7441 significantly decreased DNA-PK phosphorylation (Fig. 1*C*). Treatment with the TKI, dasatinib, reduced DNA-PK phosphorylation at 30 nM (Fig. 1*C*). Combining M3814 or NU7441 with dasatinib led to a further reduction in DNA-PK phosphorylation (Fig. 1*C*). No significant changes in total DNA-PKcs expression were observed (supplemental Fig. S1).

### KIT V560G (Juxtamembrane) and D816V (Tyrosine Kinase Domain) Mutations are Associated With Increased Sensitivity to DNA-PK Inhibitors

To investigate the therapeutic potential of inhibiting DNA-PK in KIT-mutant cells, we assessed cell survival in response to DNA-PK inhibition. M3814 is a potent specific inhibitor of DNA-PK (DNA-PK_IC50_ = 0.0006 μM) currently in clinical trial in combination with chemotherapy for relapsed/refractory AML (NCT03983824). NU7441 is an ATP-competitive inhibitor of DNA-PK (DNA-PK_IC50_  =  0.014 μM, mTOR_IC50_  =  1.7 μM); however, the low solubility profile of NU7441 restricts its use to *in vitro* applications (57). The FDC-P1 cell lines signaling through activated KIT (D816V and V560G) were more sensitive to M3814 and NU7441, compared with the EV control cells (supplemental Fig. S2).

### Inhibition of KIT Signaling is Synergistic With DNA-PK Inhibition in KIT Mutant Cells

**Fig. 2:**
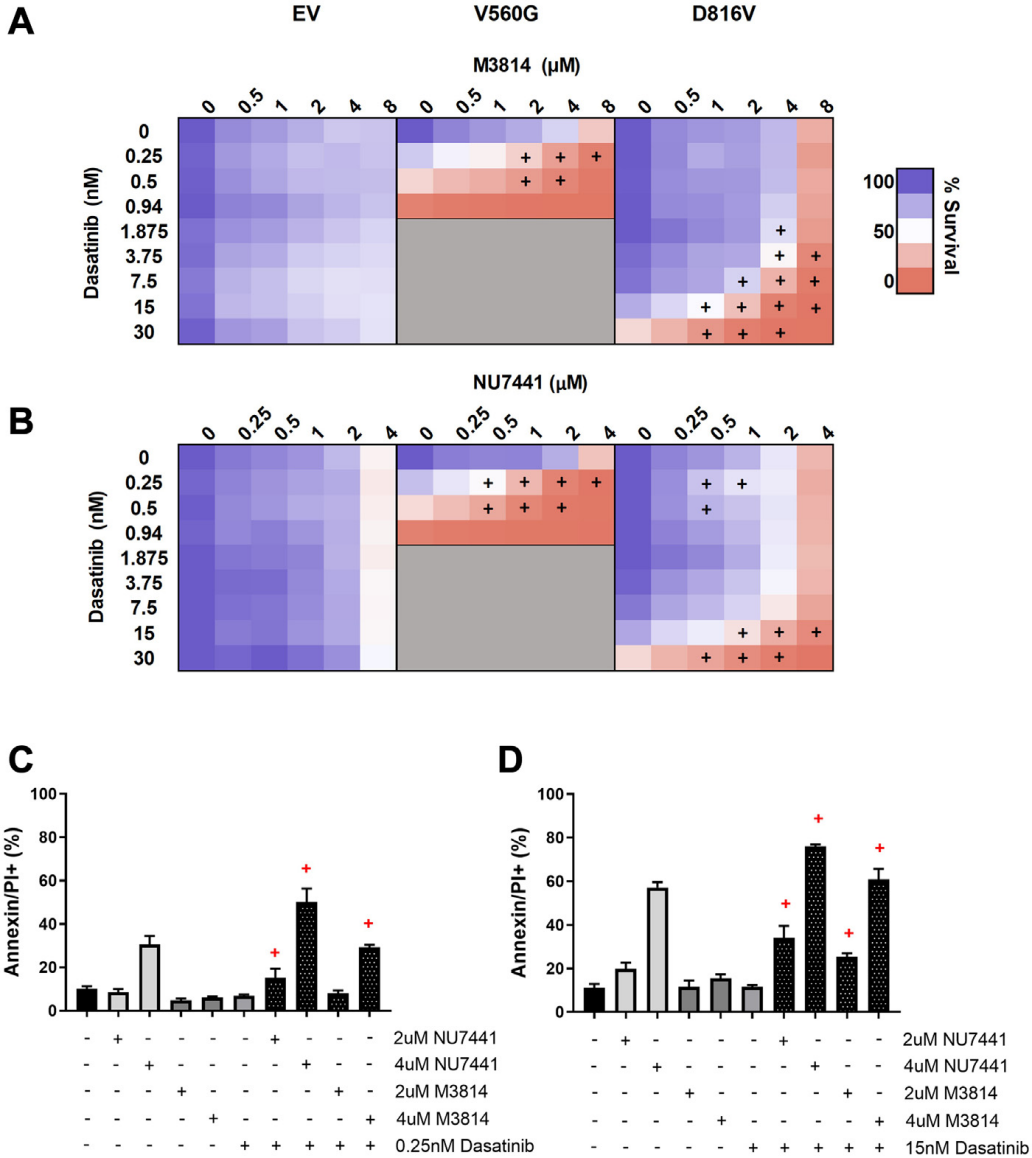
**The tyrosine kinase inhibitor, dasatinib, in combination with DNA-PK inhibitors, M3814 or NU7441, induces synergistic cell death in KIT mutant cells.** FDC-P1 cells expressing an empty vector (EV), or factor-independent mutant forms of KIT (V560G and D816V), were incubated with increasing concentrations of (*A*) dasatinib, M3814, or their combination; and (*B*) dasatinib, NU7441, or their combination. Cell viability at 72 h was assessed by resazurin metabolic assay. n ≥ 2. *Gray shading* = not done. +, synergy, assessed by the fractional product method of Webb. Apoptosis induction at 48 h in (*C*) V560G-KIT and (*D*) D816V-KIT cells was measured by annexin-V flow cytometry. Points = mean ± SEM, n ≥ 3. +, synergy, assessed by the fractional product method of Webb (supplemental Table S4). DNA-PK, DNA-dependent protein kinase; KIT, mast/stem cell growth factor receptor kit.

Subsequently, we assessed whether targeting DNA-PK in combination with inhibition of KIT signaling would lead to an enhanced antiproliferative effect in KIT-mutant cells. Dasatanib is an ATP-competitive TKI with activity against KIT, BCR-ABL, and Src family kinases (58, 59). Dasatinib is currently in clinical use for hematologic malignancies including chronic myeloid leukemia and ALL (60). As expected, the KIT mutant lines were sensitive to dasatinib treatment, with the V560G-KIT cells being more sensitive than the D816V-KIT lines, as previously reported (61); whereas dasatinib had no effect on the EV control cells (supplemental Fig. S2). The combination of dasatinib with the DNA-PK inhibitors M3814 (Fig. 2*A*) or NU7441 (Fig. 2*B*) led to a synergistic reduction in cell survival, selectively in the mutant KIT lines (V560G and D816V). To investigate the mechanism of cell death, we measured apoptosis following treatment with M3814, NU7441, dasatinib, or their combinations. The combination of either M3814 or NU7441 with dasatinib synergistically increased apoptosis, in both V560G-KIT and D816V-KIT cells (Fig. 2, *C* and *D*).

### Phosphoproteomic Analysis of Combined DNA-PK and KIT Signaling Inhibition in D816V-KIT cells Reveals Co-Operative Inhibition of ERK and mTOR Signaling

To investigate the mechanism of synergistic cell death observed with combined DNA-PK and KIT signaling inhibitors, we performed a global phosphoproteomic analysis of D816V-KIT cells untreated or treated for 1 h with 4 μM M3814, 15 nM dasatinib, or their combination, in biological triplicate. Drug doses were selected that induced no, or low, cytotoxicity as single agents but were synergistically cytotoxic when used in combination (Fig. 2, *A* and *D*). Across the 12 samples, 6892 phosphopeptides were identified (5489 unique phosphosites), with expected ratios of serine, threonine, and tyrosine sites (pS:pT:pY 89.1%:10.3%:0.6%) (supplemental Table S2).

**Fig. 3:**
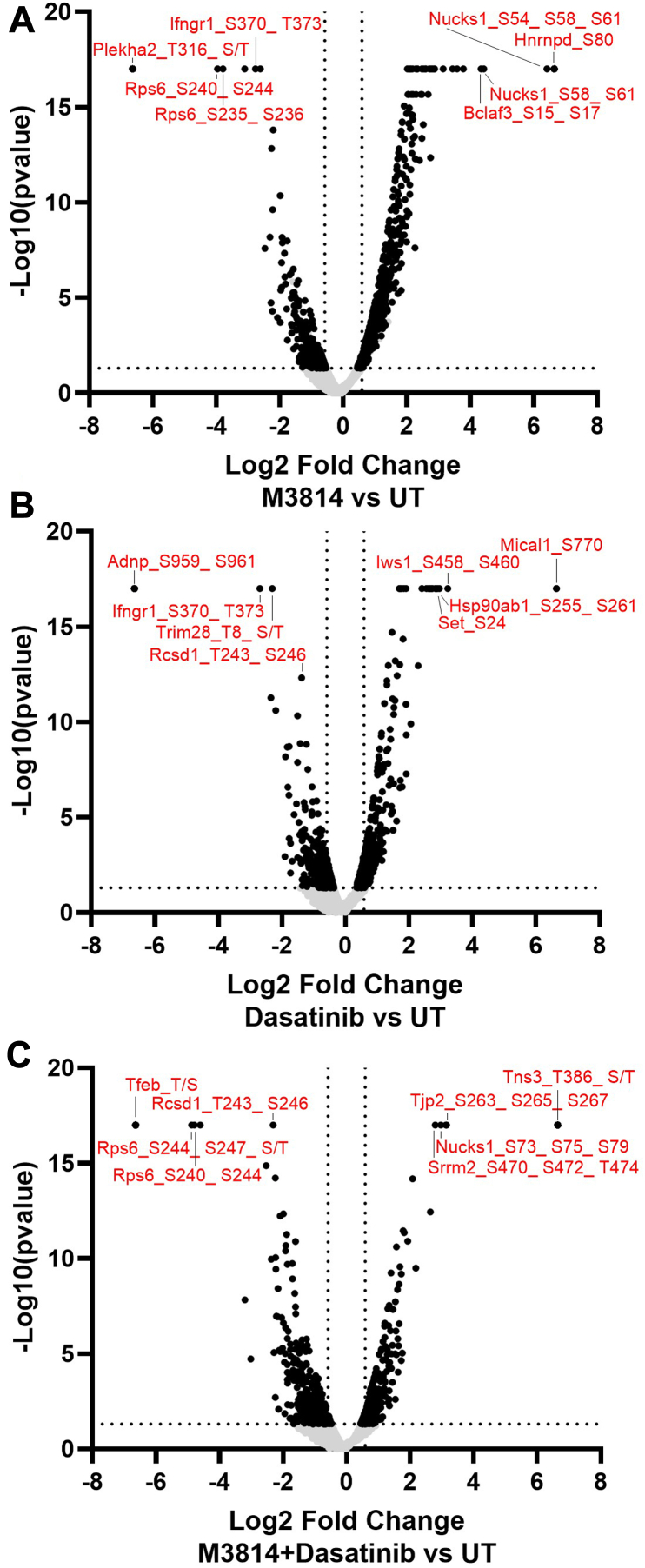
**Quantitative phosphoproteomic analysis of mutant KIT-D816V FDC-P1 cells.** FDC-P1 D816V-KIT cells were treated with M3814 (4 μM), dasatinib (15 nM), or their combination for 1 h. Phosphoproteomes were profiled by EasyPhos enrichment followed by mass spectrometry. *A*, volcano plot of significant fold changes with M3814 treatment; 616 phosphopeptides were significantly increased and 322 significantly decreased. *B*, volcano plot of significant fold changes with dasatinib treatment; 252 phosphopeptides were significantly increased and 229 significantly decreased. *C*, volcano plot of significant fold changes with combination of M3814 and dasatinib treatment; 238 phosphopeptides were significantly increased and 333 were significantly decreased. A fold-change cutoff of ±1.5 was used. KIT, mast/stem cell growth factor receptor kit.

Using a fold-change cutoff of 1.5 and *p* value <0.05, 322 phosphopeptides were decreased and 616 phosphopeptides were increased in response to M3814 treatment (Fig. 3*A*). In response to single-agent dasatinib treatment, 229 phosphopeptides were decreased and 252 were increased (Fig. 3*B*). Three hundred and thirty-three phosphopeptides were decreased, and 238 were increased in response to combination of M3814 and dasatinib treatment (Fig. 3*C*). Of these, the expression of 170 phosphopeptides was significantly modulated in all three treatment groups (supplemental Fig. S3).

**Fig. 4:**
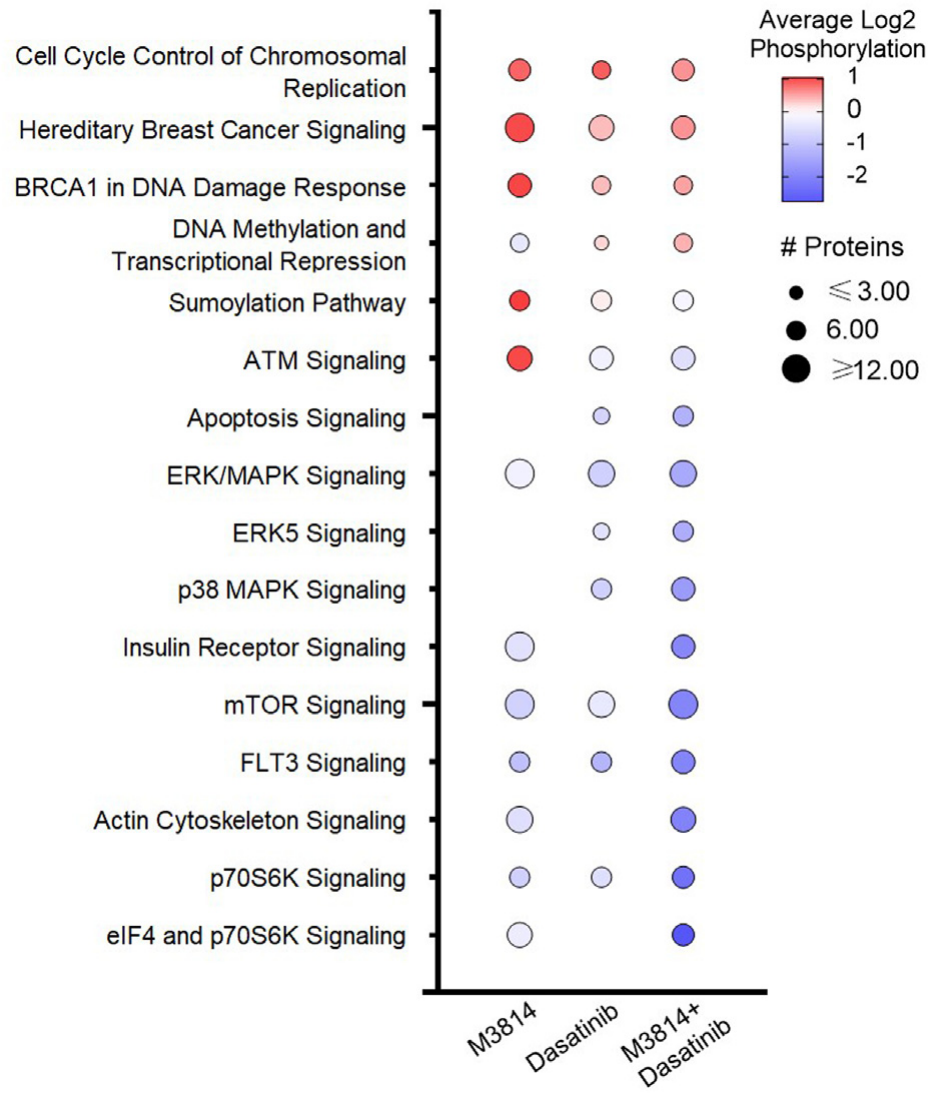
**Pathway enrichment analysis of phosphoproteins regulated by M3814, dasatinib, or their combination, in KIT-D816V FDC-P1 cells.** FDC-P1 D816V-mutant KIT cells were treated with M3814 (4 μM), dasatinib (15 nM), or their combination for 1 h. Phosphoproteins with significantly altered phosphorylation were analyzed by ingenuity pathway analysis (IPA) to identify significantly enriched pathways. Bubble size indicates number of proteins with significantly altered phosphorylation in each significantly enriched pathway, and shading indicates average pathway phosphorylation. The full list of IPA pathways is provided in supplemental Table S3. KIT, mast/stem cell growth factor receptor kit.

**Fig. 5:**
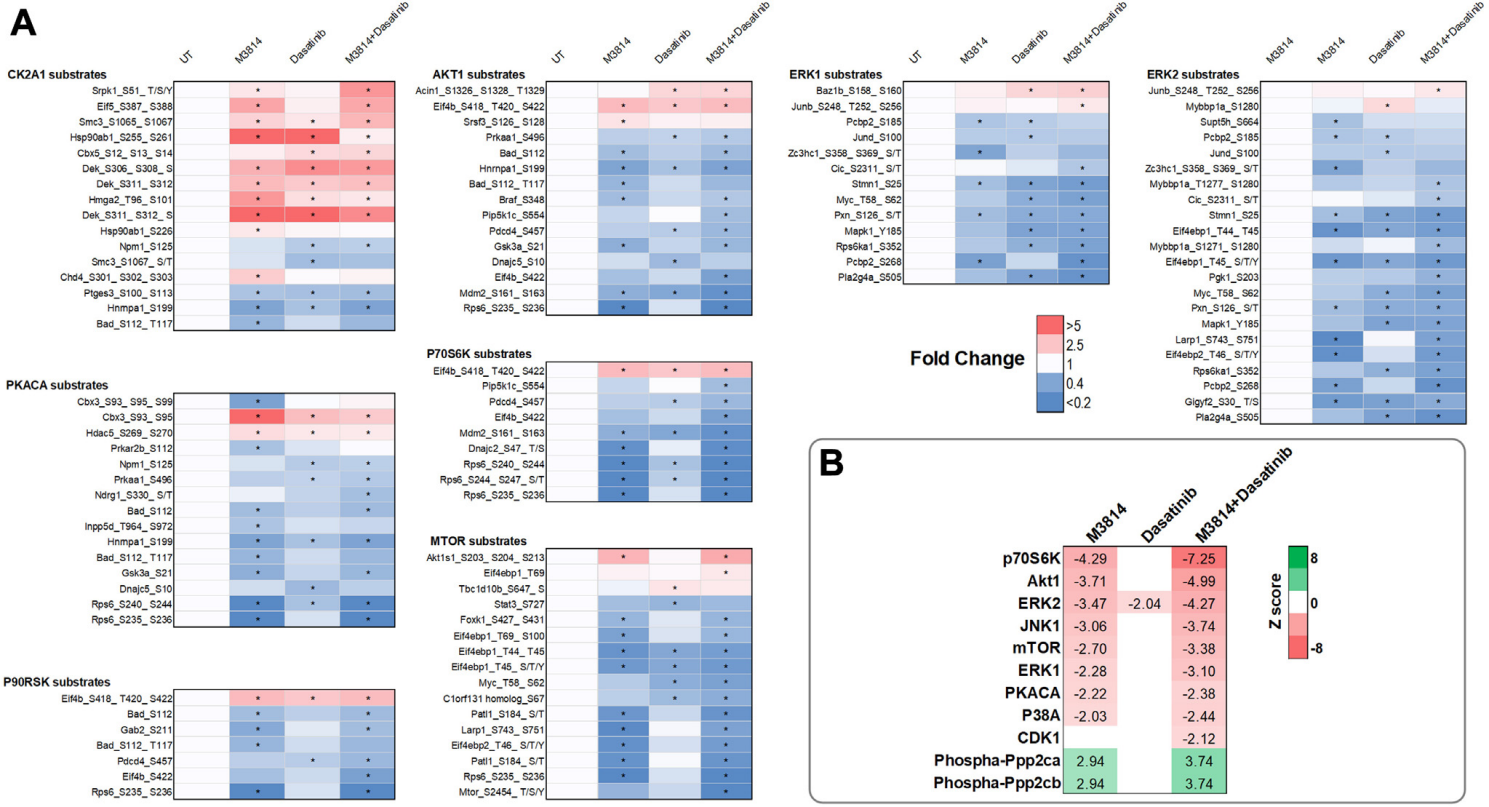
**Kinase enrichment analysis.** FDC-P1 D816V-mutant KIT cells were treated with M3814 (4 μM), dasatinib (15 nM), or their combination for 1 h. Phosphoproteomes were profiled by mass spectrometry. *A*, heatmaps of kinase substrates for kinases with more than five substrates significantly increased or decreased in phosphorylation compared with untreated (UT). ∗*p* < 0.05 compared with UT. Kinase substrates were identified using the Phosphosite Plus database (53). *B*, kinase enrichment analysis performed using RoKAI (54). Z score indicates inferred kinase or phosphatase (phospha-) activity, with a positive Z score indicating increased activity. Significant (*p* < 0.05) z scores are shown. KIT, mast/stem cell growth factor receptor kit.

Phosphopeptides with the largest fold decrease in response to M3814 single-agent treatment included RPS6 sites (pS235, pS236, pS240, and pS244) and interferon gamma receptor 1 sites (pS370 and pT373; Fig. 3*A* and supplemental Table S2). The top increased phosphopeptides included sites on NUCKS1 (pS54, pS58, and pS61) and HNRNPD (pS80; Fig. 3*A* and supplemental Table S2). Pathway analysis revealed decreased phosphorylation of mTOR, ERK/MAPK, and actin cytoskeleton signaling pathways and increased phosphorylation of BRCA1, ATM, and cell cycle pathways (Fig. 4). Concordantly, kinase enrichment analysis revealed decreased phosphorylation of P70S6K, AKT1, mTOR, ERK1/2, and PKACA substrates and increased phosphorylation of CK2A1 substrates (Fig. 5).

Phosphopeptides decreased in response to dasatinib included sites on interferon gamma receptor 1 (pS370 and pT373) and TRIM28 (pT8; Fig. 3*B* and supplemental Table S2). The top increased phosphopeptides included sites on the heat shock protein HSP90AB1 (pS255 and pS261) and transcription elongation factor IWS1 (pS458 and pS460; Fig. 3*B* and supplemental Table S2). Pathways enriched with decreased phosphorylation included p70S6K, mTOR, ERK/MAPK, p38 MAPK, and ERK5 signaling (Fig. 4). Consistent with this, phosphorylation of ERK2 substrates was decreased in response to dasatinib, including ERK2 (MAPK1) pY185; indicative of reduced ERK1/2 activity (Fig. 5). BRCA1, cell cycle, and DNA methylation and transcriptional repression signaling pathways displayed increased phosphorylation in response to dasatinib (Fig. 4).

Phosphopeptides decreased in response to the combination treatment included sites on RPS6 (pS240, pS244, and pS247) and RCSD1 (pT243 and pS246; Fig. 3*C*). The top increased phosphopeptides included sites on NUCKS1 (pS73, pS75, and pS79) and TJP2 (pS263, pS265, and pS267; Fig. 3*C* and supplemental Table S2). Pathway analysis identified decreased phosphorylation of p70S6K, mTOR, ERK/MAPK, p38 MAPK, ERK5, actin cytoskeleton, and apoptosis signaling pathways (Fig. 4). Consistent with this, phosphorylation of p70S6K, AKT1, mTOR, ERK1/2, and PKACA substrates were decreased, suggesting reduced activity of these kinases (Fig. 5). Phosphorylation of cell cycle, BRCA1, and DNA methylation and transcriptional repression signaling pathways was increased (Fig. 4), and concordantly, CDK1 substrates displayed increased phosphorylation (Fig. 5).

**Fig. 6:**
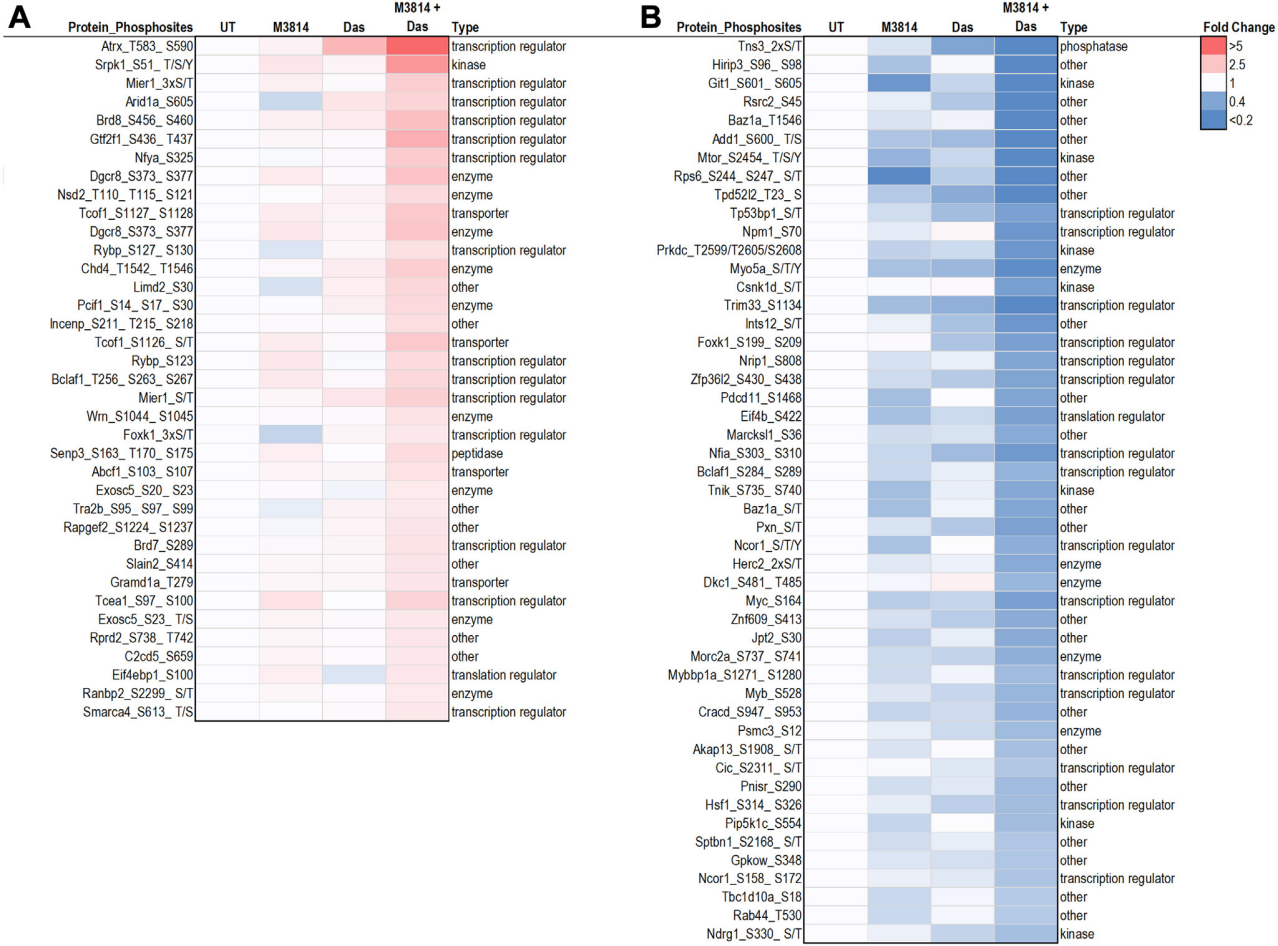
**Quantitative phosphoproteomic analysis of mutant KIT D816V FDC-P1 cells.** FDC-P1 D816V-mutant KIT cells were treated with M3814 (4 μM), dasatinib (Das; 15 nM), or their combination for 1 h. Phosphoproteomes were profiled by mass spectrometry. Heatmap of phosphosites identified with (*A*) synergistic increase or (*B*) synergistic decrease by the combination of M3814 and Das are shown. Type: ingenuity pathway analysis molecule annotations. KIT, mast/stem cell growth factor receptor kit.

**Fig. 7:**
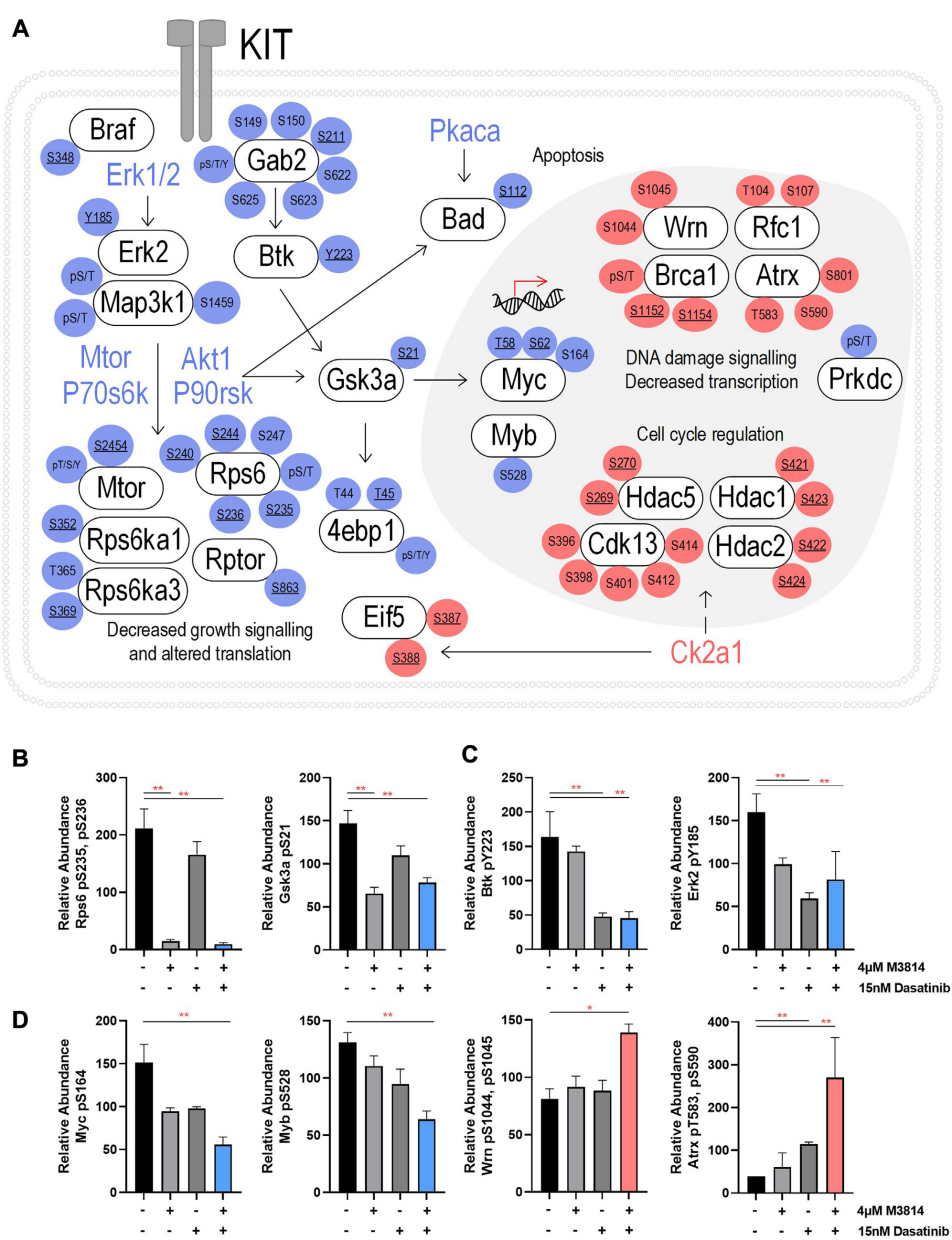
**Schematic of phosphosites increased or decreased by the combination of M3814 and dasatinib treatment, in mutant KIT D816V FDC-P1 cells.** FDC-P1 D816V-mutant KIT cells were treated with M3814 (4 μM), dasatinib (15 nM), or their combination for 1 h. Phosphoproteomes were profiled by EasyPhos enrichment followed by mass spectrometry. *A*, curation of key phosphosite changes in the identified significantly enriched pathways. Phosphosite color reflects fold change in combination treated samples compared with untreated cells (*blue* = decreased, *Red* = increased). Underlined phosphosites indicate those with annotated function (PhosphositePlus (53)). *B*, phosphosites selectively decreased by M3814 treatment. *C*, phosphosites selectively decreased by dasatinib treatment. *D*, phosphosites synergistically regulated by M3814 and dasatinib combination treatment. ∗*p* < 0.05, ∗∗*p* < 0.01. KIT, mast/stem cell growth factor receptor kit.

To investigate the crosstalk between M3814 and dasatinib treatments at the peptide level, we filtered for synergistically regulated phosphopeptides. In this context, we defined synergistic inhibition as a fold change in the M3814 + dasatinib treatment group that was ≥1.2-fold lower than the additive fold change of that in the M3814 and dasatinib single agent groups. Conversely, we defined “synergistic induction” as a fold change in the M3814 + dasatinib treatment group that was ≥1.2 higher than the additive fold change of the M3814 and dasatinib single-agent groups (62). In total, 86 phosphopeptides were synergistically regulated (37 were synergistically induced; Fig. 6*A* and 49 were synergistically inhibited; Fig. 6*B*) in response to the combined M3814 and dasatinib treatment. This included synergistic inhibition of the phosphorylation of transcription factors MYC, MYB, and NCOR1, transcription regulator NPM1, and mTOR (Figs. 6 and 7*D*). The phosphorylation of DNA repair and transcription regulators ATRX, WRN, and SMARCA4 were synergistically increased by the combination treatment (Figs. 6 and 7*D*).

Taken together, these results suggest that both M3814 and dasatinib treatment inhibit ERK/MAPK and mTOR signaling pathways, with greater inhibition of both pathways when treated in combination (Figs. 4 and 5). Both treatments increased phosphorylation of cell cycle pathways (Fig. 4), consistent with the growth arrest induced by these two inhibitors (Fig. 7*A*). The combination of M3814 and dasatinib potently inhibited both ERK/MAPK and AKT1/mTOR signaling pathways and also inhibited p90RSK kinase signaling (Figs. 4 and 5). Key phosphorylation changes in these pathways, such as RPS6 pS235, pS236, GSK3A pS21, ERK2 pY185, and BTK pY223, are driven by the effect of one inhibitor (Fig. 7, *B* and *C*). Concurrently, key phosphorylation changes such as MYC pS164 and MYB pS528 are synergistically inhibited, whereas others such as Wrn pS1044, pS1045 and ATRX pT583, pS590 are synergistically induced by the combination treatment (Fig. 7*D*).

Consistent with these results, treatment with AKT/mTOR inhibitors MK2206 and everolimus elicited synergy in combination with the MEK/ERK inhibitors, selumetinib and ASTX029 (supplemental Fig. S4). While cytotoxicity was higher in the KIT mutant lines, EV control cells were also sensitive to these combinations. PI3K/mTOR/AKT and MEK/ERK signaling pathways are involved in diverse cellular processes; therefore, it follows that targeting the activated upstream regulators, KIT and DNA-PK, elicits greater selectivity. Further supporting these findings, M3814 cotreatment with MEK/ERK inhibitors selumetinib and ASTX029 elicits synergy, with higher synergy scores compared with M3814 cotreatment with mTOR/AKT inhibitors everolimus and MK2206 (supplemental Fig. S5 and supplemental Table S5).

**Fig. 8:**
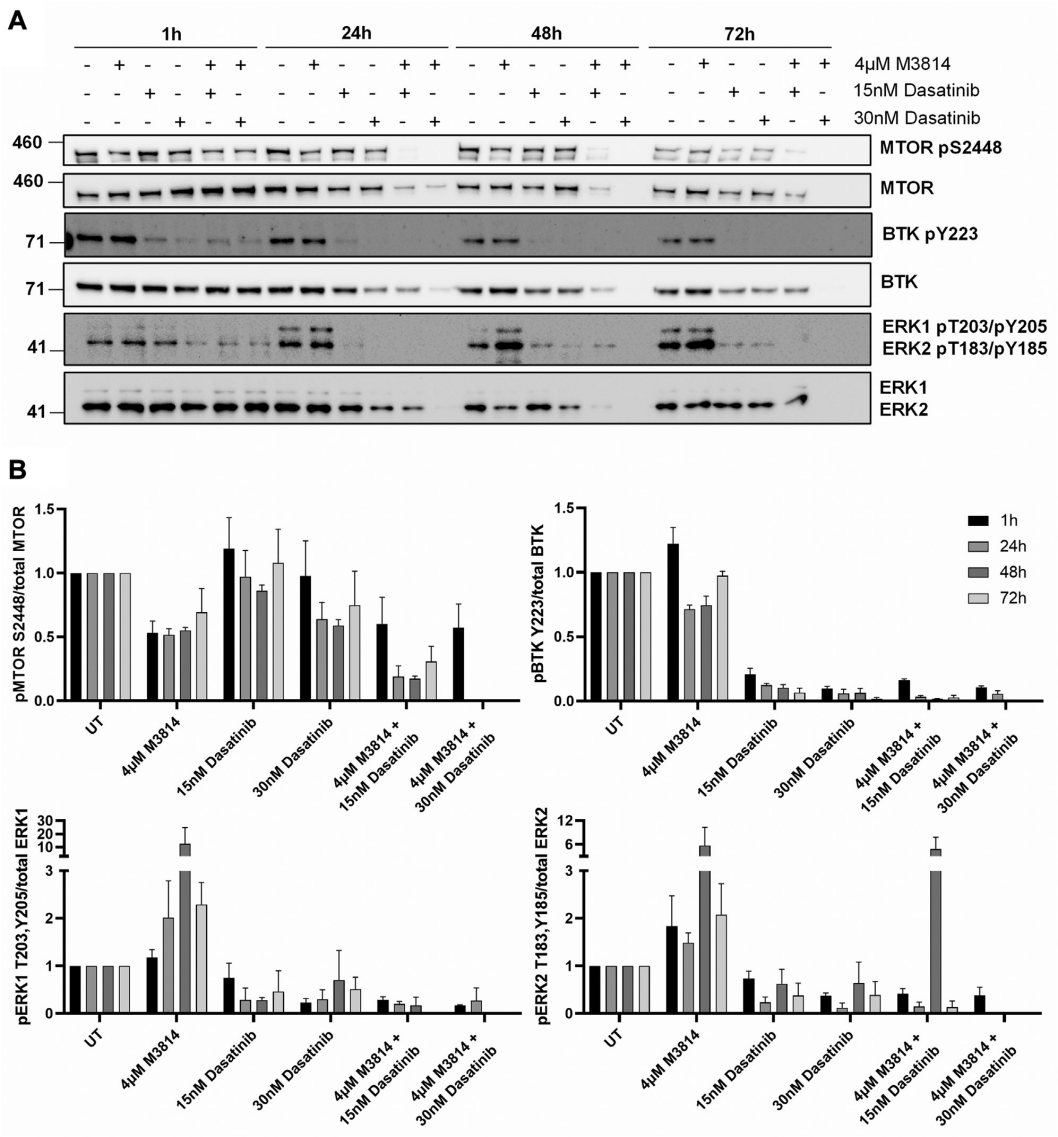
**Phosphorylation of MTOR, BTK, and ERK1/2 in response to M3814, dasatinib, or their combination.** FDC-P1 D816V-mutant KIT cells were treated with M3814 (4 μM), dasatinib (15 or 30 nM), or their combination for 1, 24, 48, and 72 h. *A*, representative Western blot images and (*B*) densitometry. Bars represent mean ± SEM. N = 2 to 3. BTK, Bruton’s tyrosine kinase; ERK1/2, extracellular signal–regulated kinase 1/2; KIT, mast/stem cell growth factor receptor kit; MTOR, serine/threonine-protein kinase Mtor.

Selected phosphoproteins were orthogonally evaluated by Western blot, over a time course of 72 h. Phosphorylation of MTOR at the mitogen-inducible phosphosite S2448 (63) was reduced by 4 μM M3814 treatment, at 1, 24, 48, and 72 h (Fig. 8). The combination of 4 μM M3814 and either 15 or 30 nM dasatinib further reduced MTOR phosphorylation at 24, 48, and 72 h. Similar to the mass spectrometry results, phosphorylation of BTK at autophosphorylation site Y223 (64) was efficiently inhibited by dasatinib treatment, at all time points (Fig. 8). Phosphorylation of ERK1/2 at the activating phosphosites T203/Y205 (ERK1) or T183/Y185 (ERK2) (65) was increased in response to M3814 treatment at later time points (24, 48, and 72 h). This effect was opposed by dasatinib, with reduced ERK1/2 phosphorylation observed in response to dasatinib both alone and in combination with M3814, excepting 15 nM dasatinib combined with M3814 at 48 h (Fig. 8).

### Inhibition of KIT Downstream Signaling Synergizes With DNA-PK Inhibitors in Mutant KIT Cells

**Fig. 9:**
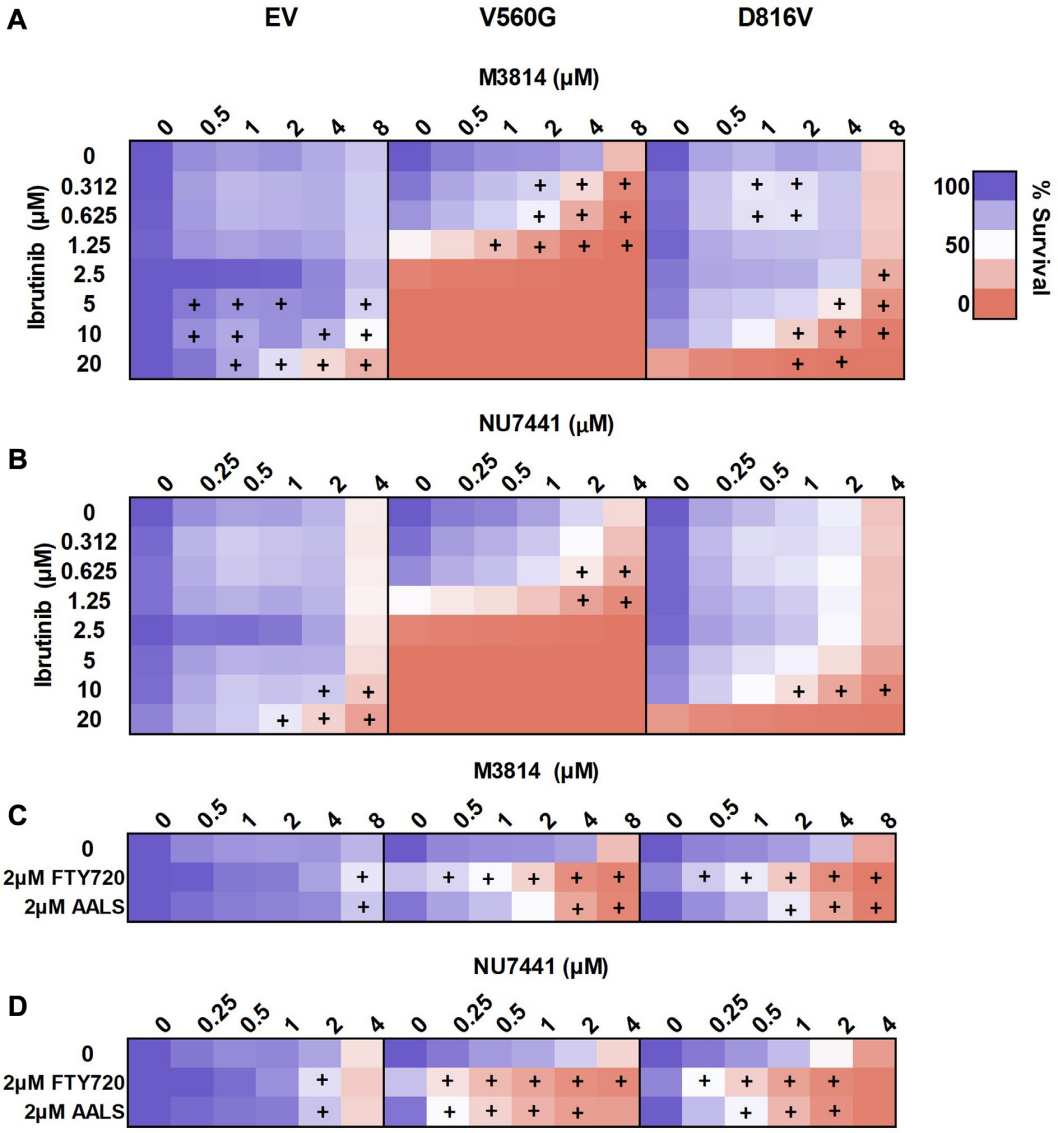
**KIT signaling inhibitors ibrutinib, FTY720, or AALS in combination with DNA-PK inhibitors NU7441 or M3814 induce synergistic cell death in KIT-mutant cells.** FDC-P1 cells expressing an empty vector (EV), or factor-independent mutant forms of KIT (V560G and D816V), were incubated with increasing concentrations of (*A*) ibrutinib, M3814, or their combination; (*B*) ibrutinib, NU7441, or their combination; (*C*) FTY720 or AALS, alone or in combination with M3814; and (*D*) FTY720 or AALS, alone or in combination with NU7441. Cell viability at 72 h was assessed by resazurin metabolic assay. n ≥ 2. +, synergy, assessed by the fractional product method of Webb (supplemental Table S4). DNA-PK, DNA-dependent protein kinase; KIT, mast/stem cell growth factor receptor kit.

D816V-KIT mutant cells displayed constitutive BTK phosphorylation, which was inhibited by dasatinib treatment (Figs. 7*C* and 8). This led us to assess cell survival in response to combined BTK and DNA-PK inhibition. Ibrutinib is a covalent inhibitor of BTK, in clinical use for treating lymphomas and chronic lymphocytic leukemia. Ibrutinib leads to inhibition of KIT signaling through BTK (66). Mutant KIT FDC-P1 cell lines (D816V and V560G) were more sensitive to ibrutinib compared with the EV control line (supplemental Fig. S2). The combination of ibrutinib and DNA-PK inhibitors M3814 (Fig. 9*A*) or NU7441 (Fig. 9*B*) effected synergistic reduction in cell survival in all cell lines, irrespective of KIT signaling dependence. However, the combination treatments were more potent in mutant KIT cell lines (D816V and V560G; Fig. 9, *A* and *B*).

Our laboratory has previously shown that oncogenic mutant *KIT* is associated with inhibition of the serine/threonine protein phosphatase PP2A (56). In wildtype cells, PP2A negatively regulates KIT-activated pathways including MAPK, JAK/STAT, and PI3K/AKT (67, 68). Activation of PP2A in KIT mutant cells leads to reduced phosphorylation of these pathways and subsequently apoptosis (56). Substrate enrichment analysis of M3814 and dasatinib-regulated phosphosites identified dephosphorylation of PP2A substrates (Fig. 5*B*). We therefore tested the interaction between the PP2A-activating compounds FTY720 or AAL(S) and DNA-PK inhibitors M3814 or NU7441. As expected, cells dependent on active KIT were more sensitive to FTY720 and AAL(S) compared with EV controls (56) (Fig. 9, *C* and *D*). DNA-PK inhibitors M3814 or NU7441, in combination with FTY720 or AAL(S), led to a synergistic reduction in cell growth in all FDC-P1 cell lines (Fig. 9, *C* and *D*). However, each DNA-PK inhibitor and PP2A activator combination was more potent in the V560G- and D816V-KIT lines, compared to the EV control line (Fig. 9, *C* and *D*).

## Discussion

Mutations in genes involved in kinase signaling represent a major proportion of recurrent mutations in AML (7, 9). These include the RTKs KIT and FLT3, and the mutational activation of these kinases is associated with poor treatment outcome and higher relapse risk (5, 33, 34, 35). Herein, we have shown for the first time that mutant KIT is associated with increased phosphorylation of the DNA double-strand break repair protein, DNA-PK. We demonstrate that DNA-PK and KIT signaling inhibitor combinations are synergistic and potent in *KIT* mutant cell lines, supporting DNA-PK as a novel target for therapy in *KIT* mutant cancers.

DNA-PK is the ubiquitously expressed (69) core kinase of the nonhomologous end joining DNA double-strand break repair pathway. The function of DNA-PK is highly regulated by phosphorylation (70), which is catalyzed by ATM (71), ATR (72), PLK1 (73), and DNA-PK itself (70). DNA-PK phosphorylation is also regulated by AKT (74); however, it is not known whether this regulation is direct. In mouse myeloid progenitor FDC-P1 cells expressing constitutively active mutant KIT, we identified increased phosphorylation of DNA-PK at T2599/T2605/S2608/S2610 (homologous to T2603/T2609/S2612 in human DNA-PK). These sites are situated within the ABCDE cluster of phosphorylation sites that are required for the DNA repair activity of DNA-PK (70), indicating that DNA-PK is active in KIT mutant cells. This work adds KIT to a list of growth factor receptors linked with altered DNA-PK activity, including FLT3 (41), EGFR (75), MPL (76), and MST1R (77). Activated growth signaling and DNA damage stress are two hallmarks of cancer, and it is possible that these characteristics are causatively linked (78).

We investigated the potential therapeutic benefit of targeting KIT signaling in combination with DNA-PK. The TKI, dasatinib, in combination with DNA-PK inhibitors (M3814 and NU7441) induced a synergistic and potent reduction in survival in KIT mutant cell lines (D816V and V560G) (Fig. 2). To investigate the cellular pathways mediating this synergy, we performed a global phosphoproteomic analysis of D816V-KIT cells treated with single-agent dasatinib or M3814 or their combination (Fig. 3). Dasatinib single-agent treatment reduced ERK2 kinase activity (Fig. 5) and decreased phosphorylation of p70S6K, mTOR, and ERK/MAPK pathways (Fig. 4). D816V-KIT expressing cells display constitutive phosphorylation of ERK and AKT, suggesting their activation (42). Dasatinib-induced apoptosis of D816V-KIT expressing cells has been previously correlated with a reduction of ERK2 phosphorylation, with higher doses of dasatinib (>100 nM) required to inhibit Akt phosphorylation (79). Concurrently, single-agent dasatinib treatment increased phosphorylation of cell cycle and BRCA1 DNA damage response pathways (Fig. 4), consistent with the induction of growth arrest (Fig. 2).

Treatment with single-agent M3814 decreased mTOR, AKT1, and ERK1/2 activity (Fig. 5). This was associated with decreased phosphorylation of mTOR, ERK/MAPK, insulin receptor signaling, and eIF4 and p70S6K signaling pathways (Fig. 4). A rebound increase in ERK1/2 phosphorylation was observed at 24, 48, and 72 h following M3814 treatment (Fig. 8). Rebound ERK activation has been observed in response to Raf and MEK inhibitors in numerous studies (80, 81, 82); however, this is the first report in response to a DNA-PK inhibitor. DNA-PK has been previously shown to phosphorylate AKT1 (serine 473) in response to insulin stimulation and DNA damage (83, 84); and through AKT-dependent signaling, DNA-PK regulates MYC stability (85). Indeed, MYC overexpressing cells are sensitive to inhibition of DNA-PK (86), suggesting a role for DNA-PK inhibitors in the treatment of MYC-dependent tumors. Consistent with this, M3814 treatment reduced MYC phosphorylation at threonine 58, serine 62, and serine 164 (supplemental Table S2); however, as a single-agent treatment, this did not reach statistical significance.

While we cannot rule out the possibility that the DNA-PK inhibitors used herein may directly inhibit mTOR, the potent inhibition of DNA-PK phosphorylation observed in response to single-agent DNA-PKi treatment (Fig. 1) suggests that on-target inhibition of DNA-PK is the major driver of the cellular response to DNA-PKi. A few lines of evidence support that DNA-PK is involved in mTOR signaling. DNA-PK was shown to be incorporated into mTORC2 complexes in keratinocytes (87). In a recent study of non–small cell lung carcinoma, DNA-PK was found to be incorporated into an alternative mTOR complex, regulating phosphorylation of mTOR downstream substrates RPS6 and 4EBP1 (88). DNA-PK activity may also be regulated by mTOR signaling, with rapamycin-induced inhibition of mTORC1 leading to increased PP2A activity, consequently increasing DNA-PK activity and AKT phosphorylation (89). Combined rapamycin and DNA-PK inhibitor treatment was required to suppress AKT phosphorylation and also decreased 4EBP1 phosphorylation in human lung cancer models (89), supporting that DNA-PK is involved in regulating mTOR signaling. In the current study, M3814 treatment decreased phosphorylation of RPTOR (mTORC1 component), RPS6, 4EBP1, and LARP1 (mTORC1 targets), and reduced AKT1 activity and actin cytoskeleton signaling (mTORC2 targets), suggestive of inhibition of both mTORC1 and mTORC2 activity. In this way, inhibition of DNA-PK may regulate protein translation, glucose metabolism, transcription, and cell growth.

M3814 single-agent treatment of D816V-KIT cells also increased phosphorylation of cell cycle, and ATM and BRCA1 DNA damage response pathways (Fig. 4). Increased phosphorylation of homologous recombination repair proteins BRCA1 and FANCM, coupled with increased phosphorylation of cell cycle kinases CDK12 and CDK13 (supplemental Table S2), is consistent with the induction of growth arrest (Fig. 2).

Combination M3814 and dasatinib treatment of D816V-KIT cells enhanced the effects of the single-agent treatments, with further inhibition of ERK1/2, AKT1, P70S6K, mTOR, and PKACA kinase activity (Fig. 5). This was associated with decreased phosphorylation of P70S6K, mTOR, ERK/MAPK, insulin receptor signaling, and actin cytoskeleton signaling pathways (Fig. 4). The combination of M3814 and dasatinib blocked the rebound increase in ERK phosphorylation observed in response to M3814 single-agent treatment (Fig. 8). This block is likely an important contributor to the synergistic cytotoxicity observed with the combination. Apoptosis signaling was enriched with the combination treatment (Fig. 4), with decreased phosphorylation of antiapoptotic proteins BAD, ROCK1, and RPS6KA1 (supplemental Table S2 and Fig. 7*A*). This is indicative of reduced antiapoptotic signaling, consistent with the synergistic induction of apoptosis effected by M3814 and dasatinib in D816V-KIT cells (Fig. 2*D*). Concurrently, increased phosphorylation of cell cycle and BRCA1 DNA damage signaling pathways (Fig. 4) and increased CDK1 activity (Fig. 5*B*) also indicate an induction of growth arrest in response to the combination treatment. Interestingly, phosphorylation of proteins annotated in ATM signaling, including CBX1, CBX3, and KAT5, were increased with M3814 single-agent treatment but not in combination with dasatinib (Fig. 4 and supplemental Table S2). Thus, it is possible that DNA-PK inhibition by M3814 treatment alone may result in concomitant activation of alternate DNA damage repair pathways, which could be prosurvival. Inhibition of these pathways with the addition of dasatinib could contribute to the synergistic cytotoxicity observed. Along with the additive phosphorylation effects of the combined inhibitors, 86 phosphopeptides were synergistically regulated by the combination treatment (Fig. 6). These phosphopeptides map to proteins regulating transcription, translation, and RNA metabolism (Fig. 6). This included a synergistic decrease in phosphorylation of transcription factors MYC, MYB, and NCOR1 (Fig. 6), suggesting a synergistic inhibition of the activity of these transcription factors. Phosphorylation of DNA repair and transcription regulators ATRX, WRN, and SMARCA4 was co-operatively increased by the combination treatment (Fig. 6), suggesting synergistic regulation of DNA damage signaling.

D816V-KIT cells displayed constitutive phosphorylation of BTK (Fig. 7), which was reduced by dasatinib treatment, alone and in combination with M3814 (Fig. 7). BTK is activated downstream of a range of receptors, including KIT (66) and Fc receptors (90, 91) and leads to signaling through PI3K, PLCy, PKC, and MAPK pathways (reviewed in Ref. (92)). This led us to assess whether DNA-PK inhibitors would synergize with the BTK inhibitor ibrutinib, in cells with activated KIT signaling. Ibrutinib is not only a covalent inhibitor of BTK but also functions as an inhibitor of FLT3 (93, 94), BLK, BMX, JAK3, and platelet-derived growth factor receptor α, among others (94, 95). Similar to the results observed with dasatinib, the V560G KIT cell line was more sensitive to single-agent ibrutinib compared with D816V-KIT cells (supplemental Fig. S2). This may be a result of V560G-KIT cells preferentially signaling through JAK-regulated pathways, whereas D816V-KIT cells preferentially signal through mTOR (96). Combining DNA-PK inhibitors with ibrutinib led to synergy in all cell lines, irrespective of KIT signaling dependence (Fig. 9). This may be due to the role of BTK in granulocyte-macrophage colony–stimulating factor signaling (97), in addition to its role in KIT signaling. However, both DNA-PK inhibitor combinations with ibrutinib were more potent in mutant KIT cell lines compared with the EV control (Fig. 9).

Finally, we showed that inhibition of KIT signaling by phosphatase activation also synergized with DNA-PK inhibition. The phosphatase PP2A negatively regulates c-KIT (56) and FLT3 (98) activated signaling pathways; but also regulates a number of cellular pathways involved in cell growth signaling, cell cycle, metabolism, and DNA repair and replication (67, 99). PP2A activators FTY720 and AAL(S), used in combination with DNA-PK inhibitors M3814 and NU7441, were synergistic in all cell lines. The PP2A activator and DNA-PK inhibitor combinations however were more potent in cell lines expressing mutant KIT (D816V and V560G; Fig. 9).

Together, our results demonstrate that cells driven by KIT signaling are selectively sensitive to a range of KIT signaling and DNA-PK inhibitor combinations, suggesting this is a class effect. Inhibitors of KIT signaling such as ibrutinib and dasatinib are currently in clinical trial for treatment of AML and are in clinical use for other cancers (60, 100). DNA-PK inhibitors have also recently entered clinical trials for AML, and solid cancers (NCT03983824 and NCT03907969), with acceptable toxicity profiles (101, 102) suggesting favorable probability of application in AML therapy. Activating mutations of KIT occur in a range of neoplasms in addition to AML, including melanoma (25), gastrointestinal stromal tumors (26), testicular seminomas (103), and mastocytosis (24). While further preclinical evaluation is required, the results herein support that treatment combining DNA-PK inhibitors and inhibitors of KIT signaling is a promising strategy for therapy of cancers with activated KIT signaling.

## Data Availability

The mass spectrometry proteomics data have been deposited to the ProteomeXchange Consortium (http://proteomecentral.proteomexchange.org) *via* the PRIDE partner repository (104) with the dataset identifier PXD030005 and DOI 10.6019/PXD030005 for global phosphoproteomics data or *via* Panorama Public (105) (https://panoramaweb.org/R7VKXL.url) for targeted PRM data (PXD030214).

## Supplemental data

This article contains supplemental data (106).

## Conflict of interest

The authors declare that they have no conflicts of interest with the contents of this article.
