# Supplementary material for: Synergistic Targeting of DNA-PK and KIT Signaling Pathways in KIT Mutant Acute Myeloid Leukemia
Source: Mol Cell Proteomics. 2023 Jan 20;22(3):100503. doi: 10.1016/j.mcpro.2023.100503 (PMC9986649; doi:10.1016/j.mcpro.2023.100503)
Supplement: Supplementary figures and legends [file mmc1.pdf]

**Synergistic targeting of DNA-PK and KIT signaling pathways in KIT mutant acute myeloid leukemia**

Heather C. Murray[1], Kasey Miller[1], Joshua S. Brzozowski[1], Richard G.S. Kahl[1], Nathan D. Smith[2], Sean J. Humphrey[3], Matthew D. Dun[1], Nicole M. Verrills[1]

**Supplementary methods and figures:**

**Supplementary methods**

**Flow Cytometry: KIT expression**

Cells were washed twice in PBA (PBS containing 0.1% BSA and 0.1% sodium azide) prior to blocking with 10% Normal Rabbit Serum in PBA for 10 minutes on ice. Cells (5x$10^5$/sample) were then incubated with either an anti-KIT antibody (clone 1DC3) or an isotype control (IB5 antibody [1]) for 1 hour on ice. After washing 3 times with PBA, samples were incubated with secondary antibody (donkey-anti-mouse, AlexaFluor 647 conjugate, Abcam ab15107) for 45 minutes on ice, prior to 3 final washes in PBA. Samples were then resuspended in FACS fix buffer (PBS containing 2% w/v glucose, 1% formaldehyde, 0.02% sodium azide) prior to sample analysis using a FACS Canto II (BD Biosciences; Franklin Lakes, NJ, USA). Data was processed using FlowJo software (TreeStar; Ashland, OR, USA).

## Supplementary figures

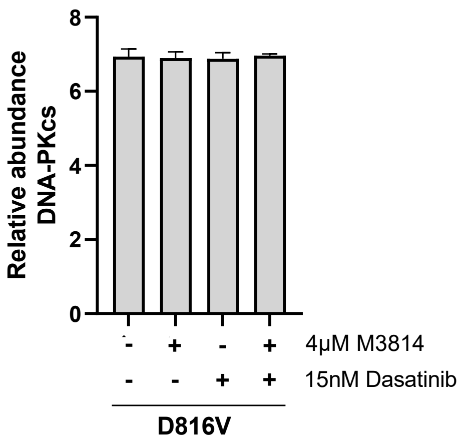


**Supplementary Figure 1: DNA-PKcs protein expression**. Protein expression of DNA-PKcs, assessed by parallel reaction monitoring (PRM) mass spectrometry, in FDC-P1/D816V-KIT cells untreated or treated for 1 h with 4μM M3814, 15nM dasatinib, or their combination. N=3 per experiment.

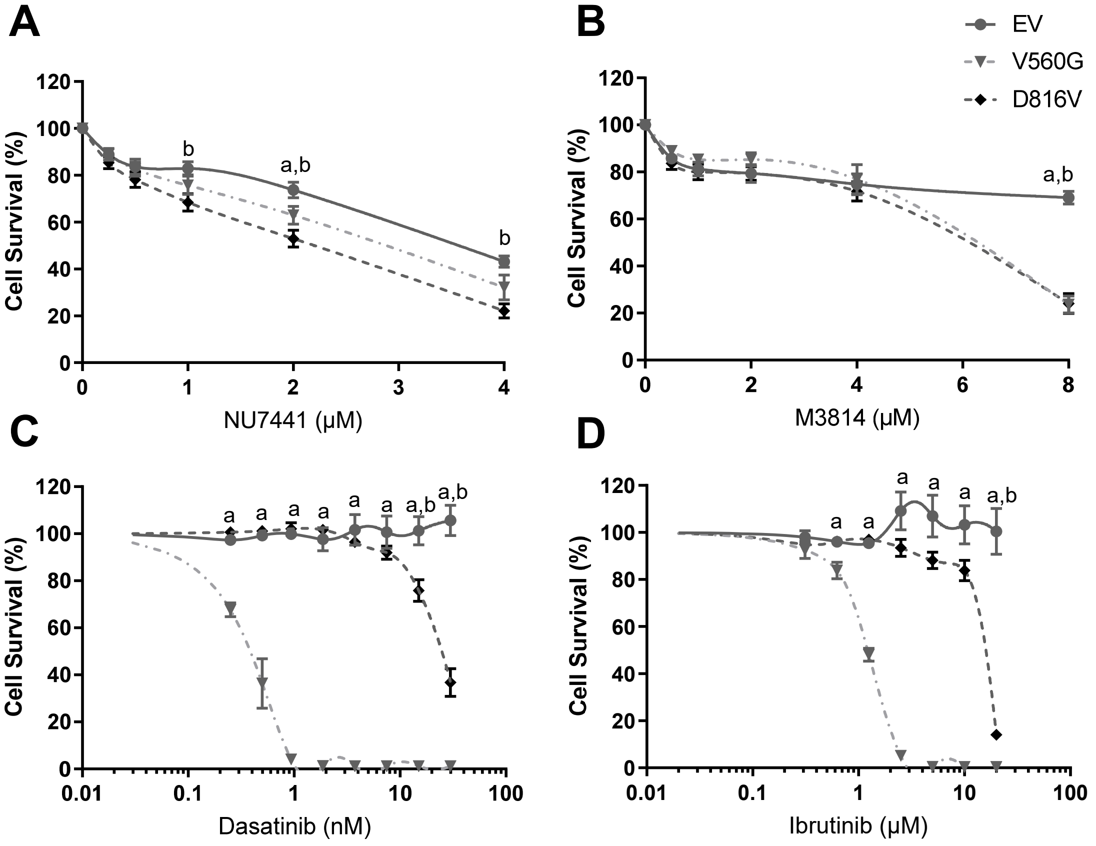


**Supplementary Figure 2: Sensitivity to DNA-PK inhibitors in FDC-P1 cells is associated with KIT-dependent growth signalling.** FDC-P1 cells were transduced with an empty vector (EV), or factor-independent mutant forms of KIT (V560G, D816V). Sensitivity to DNA-PK inhibitors A) NU7441 and B) M3814, and KIT signalling inhibitors C) Dasatinib and D) Ibrutinib was assessed at 72 hours by

resazurin assay. Points; mean ± SEM, n ≥ 2**.** a, p<0.05 (t-test) comparing EV and V560G samples. b, p<0.05 (t-test) comparing EV and V560G samples.

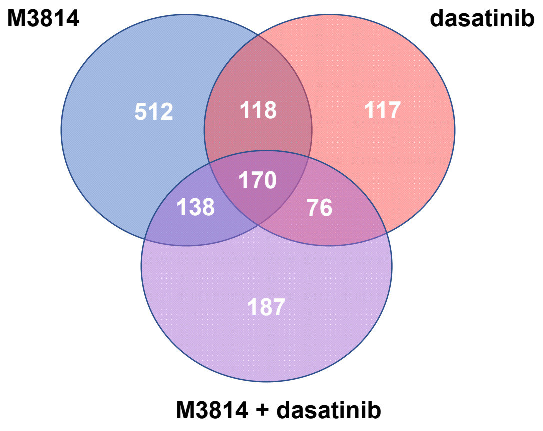


**Supplementary Figure 3: Phosphopeptides significantly altered by treatment with M3814, dasatinib, or their combination.** FDC-P1 D816V-mutant KIT cells were treated with M3814 (4μM), dasatinib (15nM), or their combination. Phosphoproteomes were profiled by EasyPhos enrichment followed by mass spectrometry.

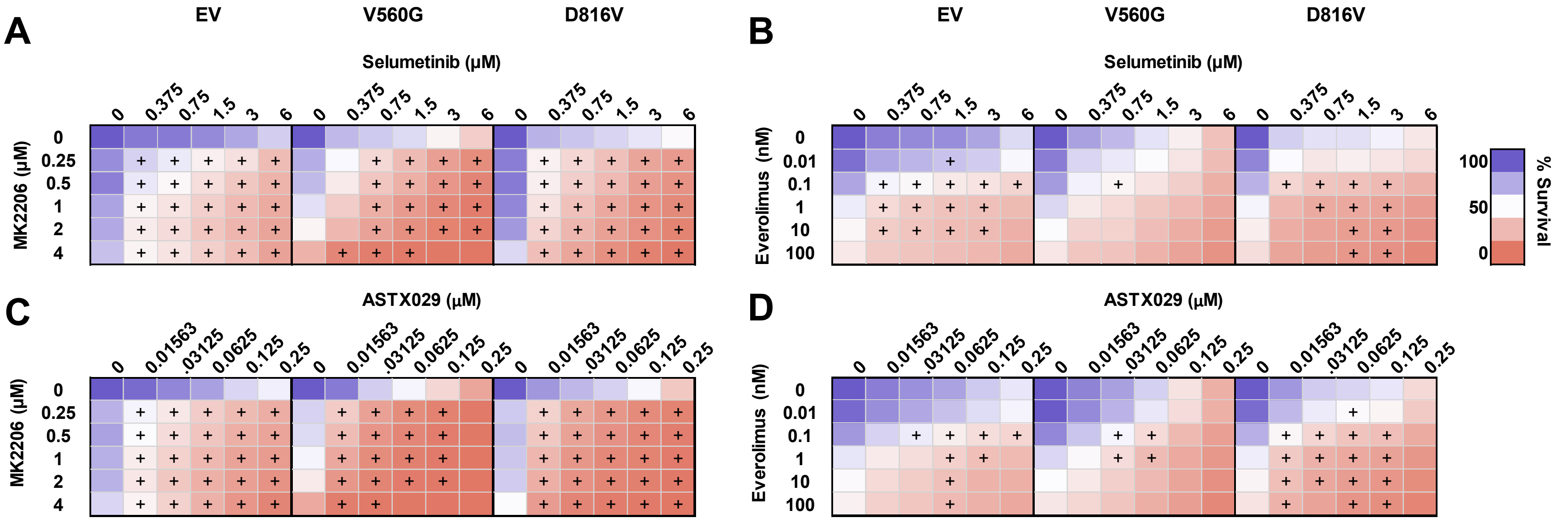


**Supplementary Figure 4: Sensitivity to AKT/MTOR inhibitors MK2206 and Everolimus, in combination with MEK/ERK inhibitors Selumetinib and ASTX029.** FDC-P1 cells expressing an empty vector (EV), or factor-independent mutant forms of KIT (V560G, D816V), were incubated with increasing concentrations of A) Selumetinib, MK2206, or their combination; B) Selumetinib, Everolimus, or their combination; C) ASTX029, MK2206, or their combination; and D) ASTX029, Everolimus, or their combination. Cell viability at 72 hours was assessed by resazurin metabolic assay. N=3. +, synergy, assessed by the fractional product method of Webb (Table S4).

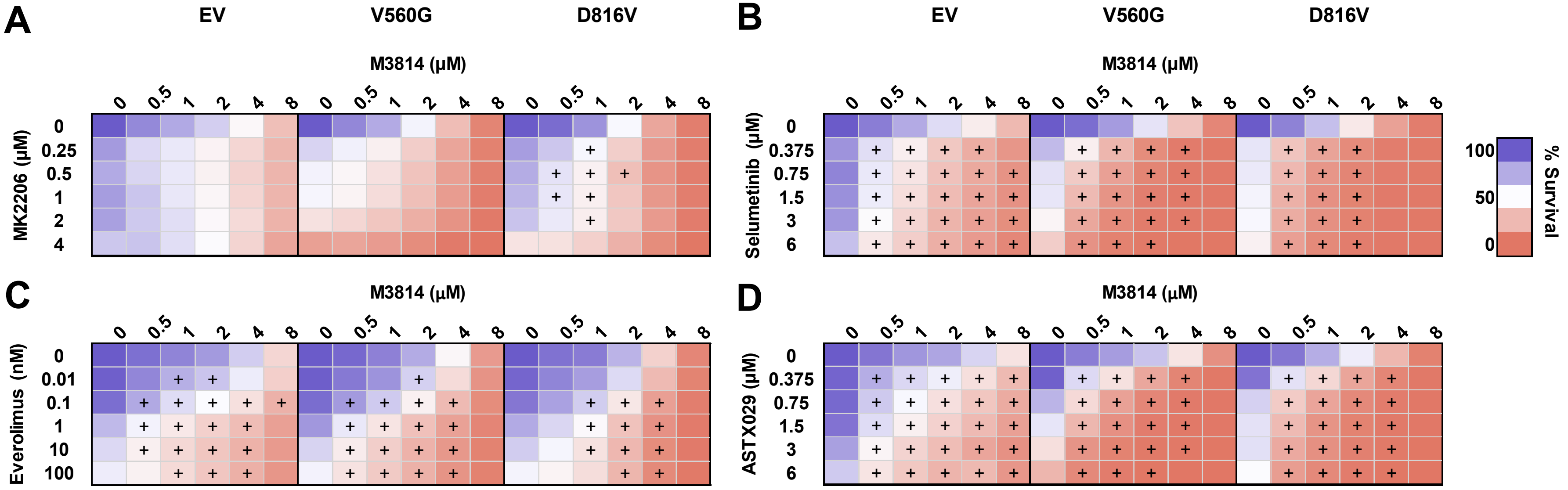


**Supplementary Figure 5: Sensitivity to AKT/MTOR inhibitors MK2206 and Everolimus, and MEK/ERK inhibitors Selumetinib and ASTX029, in combination with DNA-PK inhibitor M3814.** FDC-P1 cells expressing an empty vector (EV), or factor-independent mutant forms of KIT (V560G, D816V), were incubated with increasing concentrations of A) M3814, MK2206, or their combination; B) M3814, Selumetinib, or their combination; C) M3814, Everolimus, or their combination; and D) M3814, ASTX029, or their combination. Cell viability at 72 hours was assessed by resazurin metabolic assay. N=3. +, synergy, assessed by the fractional product method of Webb (Table S4).
